# Meta-analysis of fungal plant pathogen *Fusarium oxysporum* infection-related gene profiles using transcriptome datasets

**DOI:** 10.3389/fmicb.2022.970477

**Published:** 2022-08-24

**Authors:** Hongsheng Cai, Na Yu, Yingying Liu, Xuena Wei, Changhong Guo

**Affiliations:** Key Laboratory of Molecular and Cytogenetics, Heilongjiang Province, College of Life Science and Technology, Harbin Normal University, Harbin, China

**Keywords:** *Fusarium oxysporum*, meta-analysis, transcriptome, weighted gene co-expression network analysis (WGCNA), infection-related genes

## Abstract

*Fusarium oxysporum* is a serious soil-borne fungal pathogen that affects the production of many economically important crops worldwide. Recent reports suggest that this fungus is becoming the dominant species in soil and could become the main infectious fungus in the future. However, the infection mechanisms employed by *F. oxysporum* are poorly understood. In the present study, using a network meta-analysis technique and public transcriptome datasets for different *F. oxysporum* and plant interactions, we aimed to explore the common molecular infection strategy used by this fungus and to identify vital genes involved in this process. Principle component analysis showed that all the fungal culture samples from different datasets were clustered together, and were clearly separated from the infection samples, suggesting the feasibility of an integrated analysis of heterogeneous datasets. A total of 335 common differentially expressed genes (DEGs) were identified among these samples, of which 262 were upregulated and 73 were downregulated significantly across the datasets. The most enriched functional categories of the common DEGs were carbohydrate metabolism, amino acid metabolism, and lipid metabolism. Nine co-expression modules were identified, and two modules, the turquoise module and the blue module, correlated positively and negatively with all the infection processes, respectively. Co-expression networks were constructed for these two modules and hub genes were identified and validated. Our results comprise a cross fungal-host interaction resource, highlighting the use of a network biology approach to gain molecular insights.

## Introduction

*Fusarium oxysporum* is a common and widespread soil-borne pathogen causing diseases in over 120 plant species (Michielse and Rep, 2009). Symptoms usually comprise root rot and affected plants show stunted growth (Cai et al., 2020). As one of the top 10 most economically damaging fungal pathogens, *F. oxysporum* challenges the production of numerous economically import crops (Gordon and Martyn, 1997). In addition to causing severe losses of production, it also produces mycotoxins that pose serious threats to food safety and public health (Bani et al., 2014). Reports indicate that *F. oxysporum* is becoming the dominant pathogen in several crops, such as soybean, banana, cotton, melon, and tomato, either as destructive pathogens or potential pathogens in the near future (Swarupa et al., 2014; Bodah, 2017; Husaini et al., 2018; Cai et al., 2020). Thus, there is an urgent need to understand the mechanisms of the infection process to develop better disease prevention strategies to ensure crop yield.

Strains of *F. oxysporum* might exist saprophytically and some are considered to be non-pathogenic; however, many strains are well known to induce root rot or wilt on a variety of plants (Fravel et al., 2003). *Fusarium* spp. have complicated mechanisms to overcome plant defenses and different species might employ different infection strategies within this genus (Karlsson et al., 2021). Currently, our understanding of the infection mechanism of this pathogen is limited, yet some progress has been made. For several pathogenic strains, the secretion of various toxins is believed to play a major role in disease progression (Ramana et al., 2011). To date, the most important mycotoxins produced by *Fusarium* species from an occurrence and toxicological viewpoint are zearalenone, T-2 toxin, HT-2 toxin, fumonisins, nivalenol, and deoxynivalenol (Schelstraete et al., 2020). Genes responsible for biosynthesis of toxins and their derivatives have been identified and validated (López-Díaz et al., 2018). Besides toxins, a number of virulence genes were reported to promote the pathogenicity of *F. oxysporum* following publication of the *F. oxysporum* genome (Ma et al., 2010). Mutant screening for loss of pathogenicity of *F. oxysporum* revealed that cellular processes involving lipid metabolism and amino acids, protein translocation, cell wall integrity, and degradation of proteins seemed to be crucial for pathogenicity (Singh et al., 2017). Genes encoding effector proteins, members of the mitogen activated protein kinase (MAPK) cascade, and transcription factors were also confirmed (Taylor et al., 2016; Zuriegat et al., 2021).

RNA sequencing (RNA-seq) and microarrays are the most popular tools for functional gene identification. Compared with microarrays, RNA-Seq has low noise and high reproducibility, and allows better detection of functional genes (Kogenaru et al., 2012). Given the current low cost of sequencing, RNA-Seq has become the dominant tool. To date (October 2021), there are 51,343 series of RNA-seq experiments in the Gene Expression Omnibus (GEO) dataset located at the NCBI. This complex and large amount of data contain large volumes of raw sequencing reads produced by RNA-seq. However, these data cannot be directly interpreted by biologists, thus computational analysis and methodologies are critical to interpret the raw data to gain biological insights.

In the present study, a large scale comparative meta-analysis of RNA-seq data on *F. oxysporum* was performed. The host plants included Arabidopsis, alfalfa, soybean, bean, banana, and cucumber. The aims of this study were to: (1) Explore common genes potentially responsible for the *F. oxysporum* infection process; (2) identify candidate hub genes or key regulators in the *F. oxysporum*-plant interaction; and (3) validate candidate hub genes in biologically important modules.

## Materials and methods

### Data collection and processing

A comprehensive query for RNA-seq data from *F. oxysporum* was performed, followed by manual inspection to identify all published *F. oxysporum* and plant interaction experiments. In total, 46 published RNA-Seq samples of *F. oxysporum* from the GEO database were identified and their SRA accessions and information are listed in Supplementary Tables 1, 2. Raw RNA-seq data were downloaded from the GEO database as SRA files, and fastq-dump was used to convert SRA files into fastq data. Then, quality control was performed using FastQC to remove adapter sequences from the raw data. The preprocessed data was then mapped to the *F. oxysporum* genome using HISAT2 (van Dam et al., 2017). FeatureCounts was used to count the aligned reads (Liao et al., 2014). The merging of read counts was completed in the R software and the results were merged for each experiment. DESeq2 was used to identify the differentially expressed genes (DEGs) (Love et al., 2014).

### Kyoto encyclopedia of genes and genomes analysis of the core differentially expressed genes

The core DEGs of the infection response were identified by comparing all DEGs among different datasets using Venn diagram analysis. These genes were then subjected to Kyoto Encyclopedia of Genes and Genomes (KEGG) analysis to identify the over-represented metabolic pathways involved in the infection process.

### Weighted gene co-expression network analysis

Normalized RNA expression values [log_2_ (transcript per million (TPM) + 1)] for 9,496 DEGs (at least differentially expressed in one *F. oxysporum*-host system) were loaded into the R package weighted gene co-expression network analysis (WGCNA) (Langfelder and Horvath, 2008). The blockwiseModules function of the WGCNA package in R was used to generate modules with powers of 5, which best approximated a scale-free topology of the resultant network. Module-trait associations were estimated using the correlation between the module eigen gene and infection/control treatments. Final module colors and numbers were set as a result of this merging. Modules were exported for visualization in Cytoscape (Shannon et al., 2003) using the exportNetworkToCytoscape function in the WGCNA R package. Hub genes were identified by using cytoHubba in Cytoscape.

### *Fusarium oxysporum* culture and infection assays

*F. oxysporum* (isolated from a soybean field and stored in our laboratory) was cultured on potato-dextrose-agar for 10 days at 25°C. Spore suspensions for inoculation were prepared by flooding cultures with distilled water, and then filtering them through sterile cloth. The inoculum concentration was calculated by counting conidia in a hemacytometer under the microscope, and conidial suspensions were adjusted to a concentration of 1 × 10^6^ conidia/ml. Thirty soybean seeds of susceptible cultivar DS7 were germinated in a Petri dish with distilled water for 2 days in the dark at 25°C. Fifteen homozygous germinated seeds were transferred onto another Petri dish and inoculated individually with 200 μl of the spores. The treated seeds were incubated at 25°C in the dark. At 2 days post inoculation (2 dpi), soybean roots were frozen in liquid nitrogen and stored at −80°C.

### RNA extraction and quantitative real-time reverse transcription PCR

Total RNA was extracted from root tissues using the Trizol reagent (Invitrogen, Waltham, MA, United States) according to the manufacturer’s instructions, and the purified RNA was treated with RNase-free DNase I (Promega, Madison, WI, United States) to remove any DNA contamination. The expression patterns of selected genes were determined using quantitative real-time reverse transcription PCR (qRT-PCR) as described previously (Cai et al., 2015) using a qPCR CFX 96 Thermocycler (Bio-Rad, Hercules, CA, United States). The *EF1α* gene was used as the reference gene to normalize the relative quantification. The expression ratio and fold change (FC) were calculated using the 2^–ΔΔCt^ method (Livak and Schmittgen, 2001). All reactions were performed in triplicate. The gene-specific primers are shown in Supplementary Table 3.

## Results

### Principle component analysis of transcriptome datasets

**FIGURE 1:**
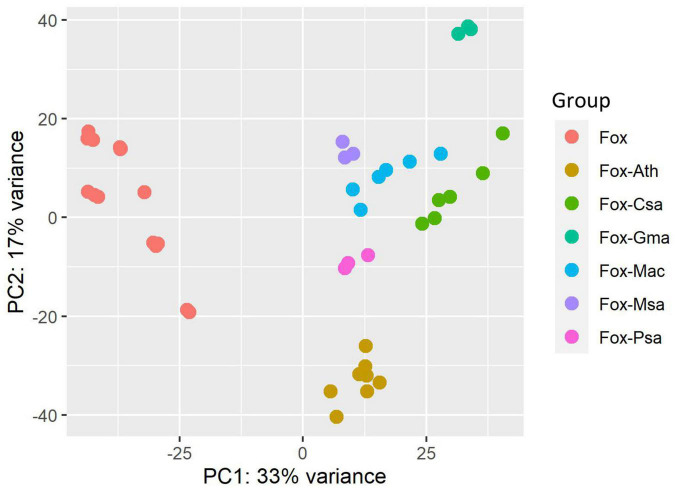
Principal component analysis (PCA) of the heterogeneous RNA-seq datasets characteristics. Fox, Fox-Ath, Fox-Csa, Fox-Gma, Fox-Mac, Fox-Msa, and Fox-Psa represent *F. oxysporum* transcriptome-wide expression profiles for an *in vitro* culture, and *F. oxysporum* infection of *Arabidopsis thaliana*, *Cucumis sativus*, *Glycine max*, *Musa acuminata*, *Medicago sativa*, and *Pisum sativum*, respectively.

The datasets used in this study were heterogeneous; therefore, we performed Principal Component Analysis (PCA) to better understand the variation among the datasets (Figure 1). PCA analysis was performed by using the plot PCA function, which is part of the DESeq2 package. Examination of the plot revealed some interesting features. First, the *F. oxysporum in vitro* culture treatments from different datasets clustered together, and were clearly separated from the infection samples by examination of Principle Component 1 (PC1). This result indicated a possible common core infection process among *F. oxysporum* strains, although with different hosts. Second, replicates within each experiment clustered relatively tightly, which indicated good reproducibility. Third, for each *F. oxysporum*-host interaction experiment, the infection samples clustered together, which indicated species-specific interactions. Together, these results indicated that there are both common core infection-related genes and also specific genes for each interaction system.

### Differentially expressed genes

**FIGURE 2:**
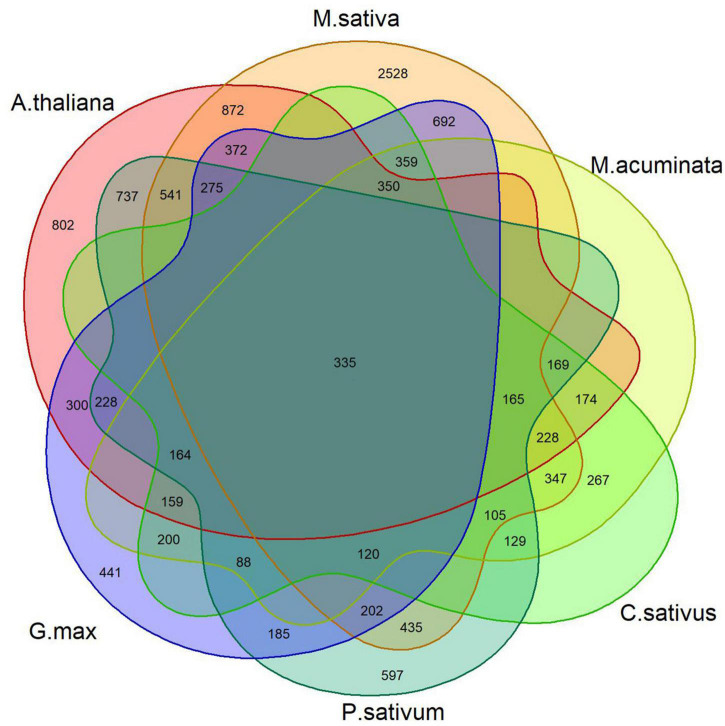
Overlap of DEGs in the *F. oxysporum* expression profile across six infected plant host examples.

For each dataset, DEGs were identified using DESeq2, which uses raw counts to perform the statistical test to identify genes that are differentially expressed between conditions. The counts of the common genes shared among individual studies are shown in a Venn diagram (Figure 2). There were 7,980, 5,925, 3,413, 7,126, 4,529, and 4,524 DEGs for alfalfa, Arabidopsis, banana, cucumber, pea, and soybean, respectively. A total of 335 common DEGs were identified among these samples. Among them, 262 were upregulated significantly, whereas 73 were downregulated across the datasets. The common DEGs, sorted by their fold-change, are shown in Supplementary Table 4.

### Kyoto encyclopedia of genes and genomes pathway analysis of common differentially expressed genes

**FIGURE 3:**
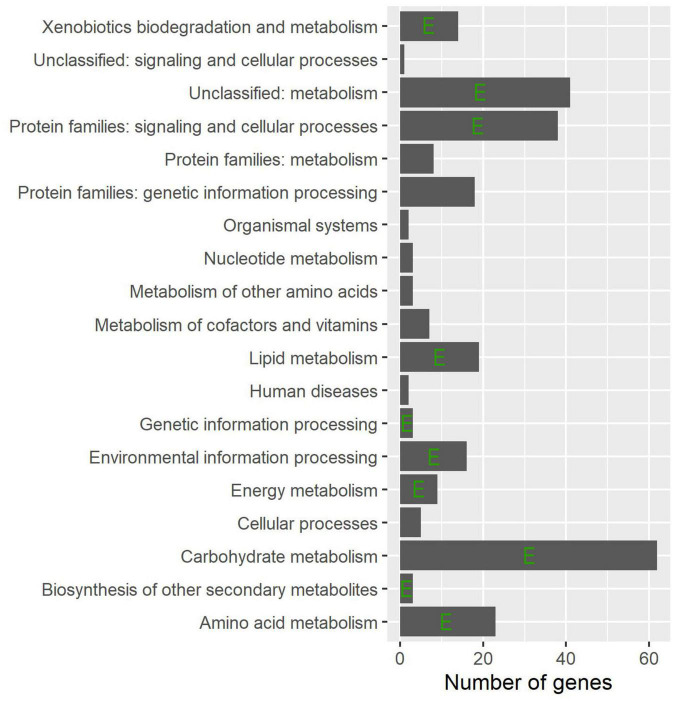
Distribution of the functional groups for the common DEGs. The horizontal columns indicate the total number of DEGs and the left axis shows their corresponding functional group names. The letter E indicates statistical significance as determined using a hypergeometric test (*P* < 0.05).

To find important pathways, pathway enrichment analysis of common DEGs was performed based on the KEGG database. The results showed that a number of metabolic pathways are involved in the common infection processes (Figure 3), e.g., carbohydrate metabolism, amino acid metabolism, and lipid metabolism. Moreover, biosynthesis of secondary metabolites was also significantly enriched. As expected, environmental and genetic information processing were also enriched.

### Weighted gene co-expression network analysis

**FIGURE 4:**
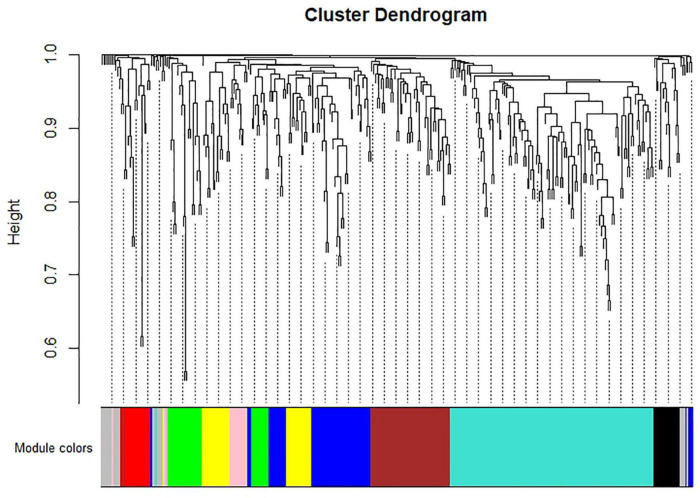
Hierarchical cluster tree of identified co-expression modules identified by WGCNA. The major tree includes nine modules according to calculation of eigen genes. Each module is highlighted in a designated color.

**FIGURE 5:**
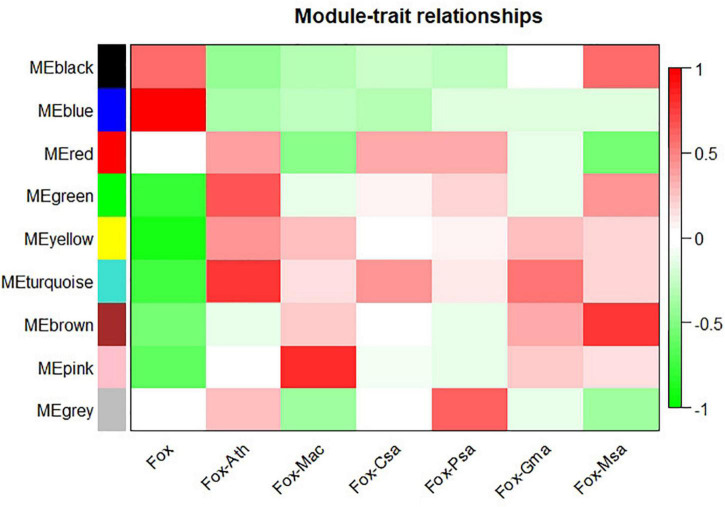
Module-sample relationships. Each row corresponds to a different module shown on the left side, and each column represents a sample. Red represents high expression, and blue represents low expression. Fox, Fox-Ath, Fox-Csa, Fox-Gma, Fox-Mac, Fox-Msa, and Fox-Psa represent *F. oxysporum in vitro* culture, and *F. oxysporum* infection of *Arabidopsis thaliana*, *Cucumis sativus*, *Glycine max*, *Musa acuminata*, *Medicago sativa*, and *Pisum sativum*, respectively.

WGCNA analysis on the gene expression profiles were performed to understand gene relationships. The gene co-expression network was constructed on the basis of the correlation between the expression values of all the sample DEGs. The soft threshold power value was set as 5, which maximized *R*^2^ while maintaining a high mean number of connections. Highly related genes formed a module, and nine co-expression modules were finally identified (Figure 4). The association of different modules with infection traits showed that the turquoise module correlated positively with all the infection processes. By contrast, the blue module correlated negatively with all the infection processes (Figure 5). The high correlation of the genes in these two modules with infection traits suggested their common involvement in *F. oxysporum* infection.

### Identification of hub genes mediating the common *Fusarium oxysporum* infection process

**FIGURE 6:**
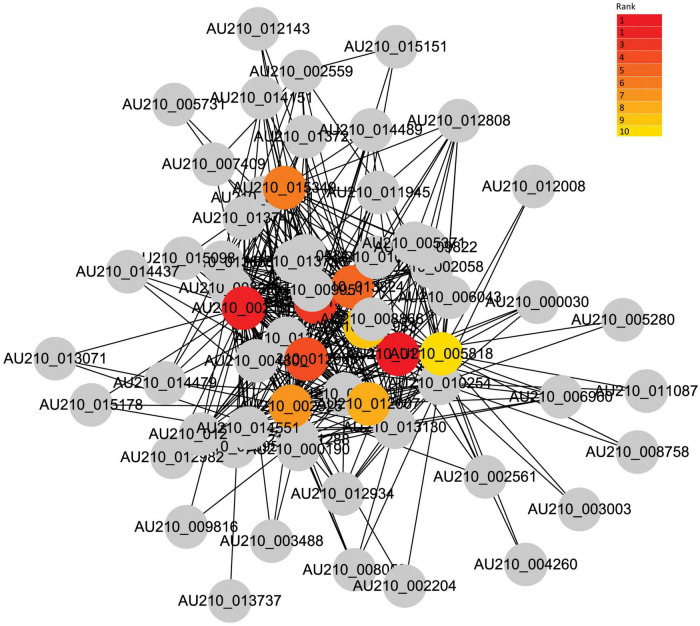
Co-expression network analysis of the turquoise module. The red to yellow circle represent the hub gene identified by cytoHubba in Cytoscape, and the color shade indicates the correlation.

**FIGURE 7:**
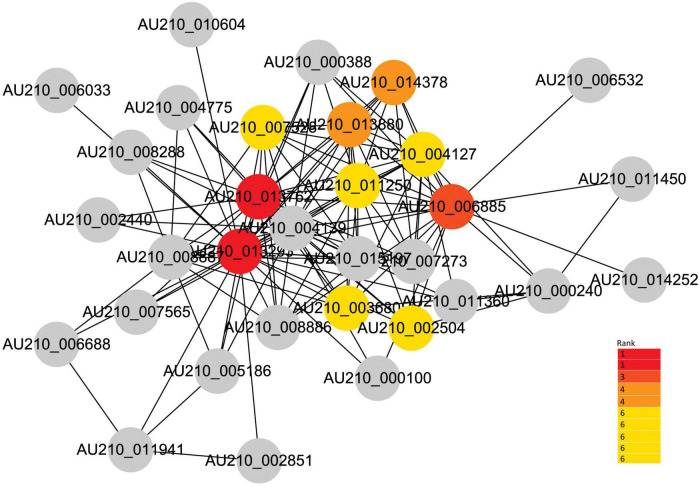
Co-expression network analysis of the blue module. The red to yellow circles represent the hub genes identified by cytoHubba in Cytoscape, and the color shade indicates the correlation.

Given the strong association of the turquoise and blue modules with the common infection process, the DEGs in these modules were exported to the Cytoscape software to obtain a co-expression network. The threshold of the weighted edges was set as 0.28 and the top ten hub genes for each module were identified using the cytoHubba plugin in Cytoscape (Figures 6, 7).

### Validation of selected hub genes

**FIGURE 8:**
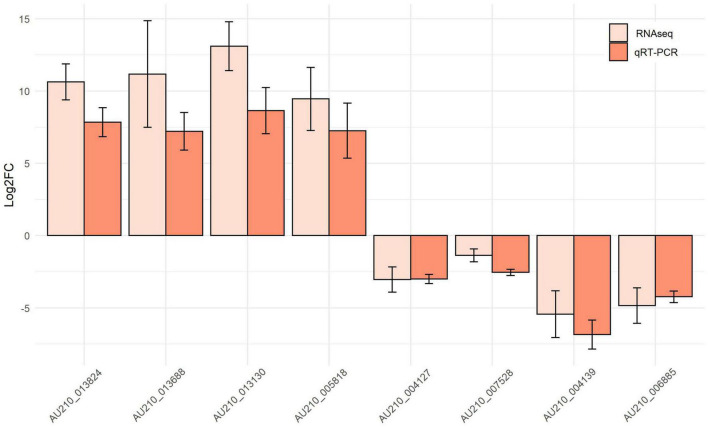
Expression patterns of selected top hub genes from the turquoise and blue modules. The expression values for each gene were obtained from the meta-summary calculations and qRT-PCR.

Hub genes in each module identified in the RNA-seq datasets were validated using qRT-PCR from samples of *F. oxysporum* infecting soybean (Figure 8). The analysis was carried out for the following top four genes in each module: AU210_011290, AU210_013824 (encoding xyloglucanase), AU210_013688, AU210_013130 (encoding proteinase); and AU210_004127, AU210_007528, AU210_004139, and AU210_006885. Limited annotation information meant that the function of most of these genes were uncharacterized. Hub genes from the turquoise module were upregulated in the infection process, whereas genes from the blue module were downregulated. Overall, the qRT-PCR analysis confirmed the results obtained from the transcriptome analysis.

## Discussion

Several studies on the distribution of *Fusarium* spp. around the world suggest that climate is a major factor in determining the distribution of these fungi in soil, which are becoming potential threats to crop yield (Doohan et al., 2003; Arias et al., 2013). To better manage this pathogen, integrated bioinformatic methods would play key roles in the functional characterization of the genes responsible for infection. The availability of the next generation sequencing technologies makes RNA-Seq affordable to study different species to answer many questions in biology. The objectives of this study were to identify common infection-related genes in *F. oxysporum* and to characterize the function of these genes.

Root rot caused by *F. oxysporum* is a common and widespread disease worldwide. Based on their host range, strains with the same plant host range are grouped into a *forma specialis* (Edel-Hermann and Lecomte, 2019). It is estimated that 106 *formae speciales* have been clearly described within *F. oxysporum*, and some of these have more than two races (Edel-Hermann and Lecomte, 2019). Despite their genetic diversity and host ranges, we hypothesis that *F. oxysporum* strains might have a common infection strategy. PCA analysis of RNA-seq datasets confirmed this hypothesis, because *in vitro* cultured *F. oxysporum* RNA expression profiles were clustered together, whereas RNA expression profiles from *in planta* infection trials were clearly separated from *in vitro* culture in PC1. Indeed, pathogenicity chromosomes were identified in the Fusarium genome, and experimental transfer of pathogenicity chromosomes from *F. oxysporum* into a non-pathogen transformed the latter into a pathogen (Ma et al., 2013). Moreover, a common infection strategy of all types of pathogens to manipulate essential mechanisms was reported (Durmuş Tekir et al., 2012). In our study, there are also specific genes involved in different pathogen-host systems, which might be responsible for host specificity.

The basic metabolic pathways are evolutionarily conserved in most fungal pathogen; however, there are species differences (Ene et al., 2014). Although the role of metabolism in host–fungus interactions is recognized, more and more studies have been devoted to further understanding the infection mechanism and the potential application for managing pathogens (Chen et al., 2019). A total of 335 common DEGs were identified in the six *F. oxysporum*-host interaction systems, most of which were enriched in the basic metabolic pathways, e.g., amino acid metabolism. Interestingly, unclassed metabolism and other secondary metabolisms were also enriched, which reflected the metabolic flexibility of *F. oxysporum* pathogenicity.

Traditional DEGs analysis from RNA-seq data is an important strategy to identify genes involved in certain processes. Although it provides large amounts of information, many irrelevant DEGs are included, which make it difficult to extract key genes. WGCNA is useful to identify the modules of co-expressed genes that are correlated with certain traits and consequently, biological pathways (Langfelder and Horvath, 2008). We identified nine modules that were related to the infection process in different host-pathogen systems. Strong positive and negative correlations of the turquoise and blue modules in all infection stages were observed. These results suggested that key genes in the above modules might play vital roles of *F. oxysporum* infection.

A protein-protein interaction network was constructed to identify key genes in the interesting modules, i.e., the turquoise and blue modules. Top hub genes for each module were identified. *F. oxysporum* genome annotation is limited; therefore, most of identified genes have unknown functions. In the turquoise module, AU210_013824 was one of the top genes identified, encoding a xyloglucanase, which is a xyloglucan-degrading enzyme. Xyloglucan is a hemicellulose that occurs in the primary cell wall of all vascular plants, and it functions as a physical barrier that separates challenging pathogens from the internal contents of the plant (Choi et al., 2013). Previous studies showed that the XEG1 protein, which exhibits xyloglucanase activity in *Phytophthora sojae*, acts as an important virulence factor during *P. sojae* infection (Ma et al., 2015). Other hub genes with known functions encoded an alkaline proteinase, an alcohol oxidase, and a beta-glucosidase. Thus, this approach identified key *F. oxysporum* infection-related genes and also a number of uncharacterized genes that have not been reported in previous studies.

Similar qRT-PCR-determined expression patterns of selected hub genes to those from the RNA-seq data were observed, which reflected the reliability of the RNA-seq data and the integrated meta-analysis. Co-expression network analysis is a biologically driven data analysis technique that has been used extensively to extract modules and genes that function in certain biological pathways. Many genes playing vital roles in multiple biological processes or molecular functions have been successfully identified using this methodology (Filho et al., 2021; Zainal-Abidin et al., 2022). In the present study, two modules that were closely related to the *F. oxysporum* infection process in all pathogen-host systems were identified and their hub genes were validated. Altogether, the results of the present study provided information about the common molecular behavior of *F. oxysporum* infection. In the future, as more RNA-seq data are generated and precise genome annotation becomes available, a clearer picture of the *F. oxysporum* infection process will be revealed.

## Data availability statement

The original contributions presented in this study are included in the article/Supplementary material, further inquiries can be directed to the corresponding authors.

## Author contributions

HC conceived and designed the experiments. NY and YL performed the experiments. HC, NY, XW, and CG analyzed the data and wrote the manuscript. All authors read and approved the final manuscript.
